# Syndrome de la selle turcique vide: à propos d’un cas

**DOI:** 10.11604/pamj.2019.33.317.17423

**Published:** 2019-08-21

**Authors:** Benilde Bepouka Izizag, Aaron Ngandu, Daddy Liombo Mbiso

**Affiliations:** 1 Service de Médecine Interne, Clinique Rapha, Kinshasa, République Démocratique du Congo; 2 Département de Médecine Interne, Cliniques Universitaires de Kinshasa, Kinshasa, République Démocratique du Congo; 3 Service d’Imagerie Médicale, Cliniques Universitaires de Kinshasa, Kinshasa, République Démocratique du Congo

**Keywords:** Syndrome, selle turcique, vide, Syndrome, sella turcica, empty

## Abstract

Le syndrome de la selle vide est une affection dans laquelle la selle turcique est partiellement ou complètement remplie de liquide céphalorachidien (LCR), entraînant un déplacement de l'hypophyse. Nous rapportons le cas d'un patient obèse de 49 ans qui a présenté de façon progressive les céphalées, une asthénie physique et une hypothyroïdie et chez qui le scanner cérébral a été en faveur d'un syndrome de la selle turcique vide.

## Abstract (en)

Empty sella syndrome is a condition in which the sella turcica is partially or totally filled with cerebrospinal fluid causing a displacement of the pituitary gland. We here report the case of a 49-year old obese patient with progressive headaches, physical asthenia and hypothyroidism. Brain scanner showed empty sella syndrome.

## Introduction

Le syndrome de la selle turcique vide (SSTV) est un trouble impliquant la selle turcique qui est une structure osseuse située à la base du cerveau et entourant la glande pituitaire [1]. Le syndrome de la selle vide est une affection dans laquelle la selle turcique est partiellement ou complètement remplie de liquide céphalorachidien (LCR), entraînant un déplacement de l'hypophyse [2]. Nous rapportons le cas d'un patient qui présente une hypothyroïdie révélant un syndrome de la selle turcique.

## Patient et observation

**Figure 1:**
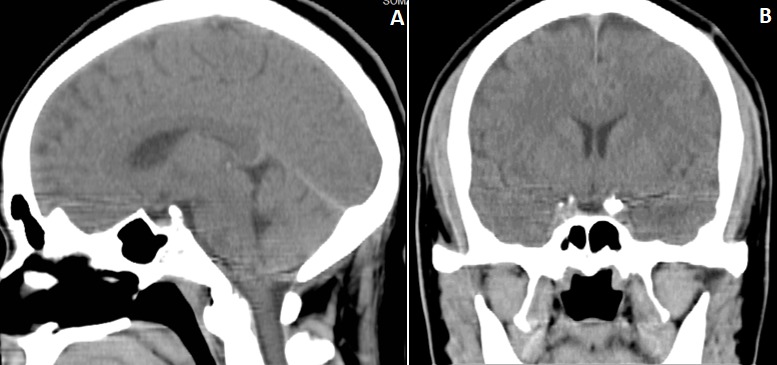
TDM cérébrale en fenêtre parenchymateuse coupes sagittale (A) et coronale (B): présence d’une hypodensité dans la loge sellaire ressemblant à l’hypodensité du 3^ème^ ventricule, le tissu hypophysaire résiduel plaqué contre le plancher sellaire traduisant donc la présence du LCR dans la loge: SSTV

Patient âgé de 49 ans qui consulte pour céphalées, des troubles visuels sous forme de vision floue, une asthénie physique et une sensation des crampes à l'hémicorps gauche évoluant depuis un mois. Dans ses antécédents nous notons qu'il est marié et père de 4 enfants. A l'examen physique, le patient a un bon état général, une pression artérielle à 122/72 mmHg, une fréquence cardiaque à 64 battements par minute, un indice de masse corporel à 30 kg/m^2^; le reste de l'examen est normal. Le bilan ophtalmologique est normal. L'échographie cardiaque, l'électrocardiogramme (ECG) et la radiographie du thorax sont normaux. Les explorations de laboratoire incluant l'hémogramme, la fonction rénale, la fonction hépatique, les électrolytes sont dans les normes. L'exploration hormonale révèle une hypothyroïdie tandis que les gonadotrophines, le cortisol, la prolactine et l'hormone de croissance sont dans les normes. L'image tomodensitométrique a révélé une vacuité de la loge de la selle turcique avec petitesse des ventricules latéraux, l'absence de toute lésion malformative de la ligne médiane, du cortex ni de la fosse postérieure (Figure 1). Le traitement de substitution à la L-Thyroxine (50μg/l) entraine la disparition de l'asthénie physique et des crampes de l'hémicorps gauche. Bilan hormonal: TSH: 7,45 (VN: 0,55-4,78); T3: 3.07 pg/ml (2.30-4.20); T4: 0.75 pg/ml (0.93-1.70); ACTH: 53,09 (VN: 7.20-63.3); LH: 4,78 mUI /ml (VN: 4.04-15.20); Prolactine: 10.40 (VN: 4.04-15.20); GH: 0.032 ng /ml (VN: 0.030-2.47).

## Discussion

La selle turcique vide (STV) est une hernie à travers le diaphragme sellaire de l'arachnoïde suivie de l'espace sous-arachnoïdien supra-sellaire, rempli de liquide céphalorachidien, dans la selle turcique [3]. Le terme STV est incorrect car en réalité la selle turcique n'est pas vide. Elle est plutôt complètement rempli par l'hypophyse, sa tige, l’arachnoïde, le LCR et parfois le système optique et le troisième ventricule. C'est pourquoi certains auteurs préfèrent utiliser le terme arachnoidocèle intrasellaire car il exprime clairement cette entité [4]. Il existe deux types de SSTV: primaire et secondaire. Le SSTV primaire survient suite à un petit défaut anatomique au-dessus de l'hypophyse qui augmente la pression dans la selle turcique et provoque l’aplatissement de la glande le long des parois intérieures de la cavité de la selle turcique. Le syndrome primaire se rencontre généralement chez les femmes d’âge moyen, obèses et hypertendues. Le trouble peut être un signe d’hypertension intracrânienne idiopathique [5]. Parmi les facteurs de risque il y a l'obésité chez la femme et la multiparité. L'obésité entraine l'apnée du sommeil obstructive avec une hypercapnie qui peut augmenter la pression du LCR et prédisposer au SSTV [6]. Ceci peut être le cas de notre patient bien qu'il soit de sexe masculin. Le SSTV secondaire résulte de la régression de l'hypophyse dans la cavité après une blessure, une chirurgie ou une radiothérapie. Les personnes atteintes de SSTV secondaire en raison de la destruction de l’hypophyse présentent des symptômes qui traduisent la perte des fonctions hypophysaires, telles que la cessation des menstruations, la stérilité, la fatigue et l’intolérance au stress et aux infections [7]. La selle turcique vide peut se traduire par plusieurs signes cliniques. Les céphalées, classiquement fronto-orbitaires, sont présentes dans 50% des cas, mais la responsabilité de la selle turcique vide est discutée par de nombreux auteurs [6-8]. Notre patient présentait aussi des céphalées frontales.

La fréquence des troubles visuels varie selon les séries mais semble peu élevée: l'altération du champ visuel (hémianopsie bitemporale voire quadranopsie bitemporale supérieure) est le symptôme le plus fréquent; un œdème papillaire voire une atrophie optique sont plus exceptionnels [8]. Notre patient présentait un flou visuel mais l'examen ophtalmologique n'a rien révélé de particulier. Les signes endocriniens ne sont pas exceptionnels et peuvent se présenter sous forme de syndrome d'hyposécrétion hormonale ou d'hypersécrétion hormonale. Le syndrome d'hyposécrétion hormonale ou hypopituitarisme, souvent partiel est retrouvé dans 15 à 30% des syndromes de selles turciques vides primaires. Parfois, les tests hormonaux dynamiques peuvent être discrètement perturbés: absence de stimulation de la fonction thyréotrope ou corticotrope ou, le plus souvent, somatotrope. Le pan hypopituitarisme est très rare [6]. Dans notre cas, le patient avait une insuffisance thyréotrope bien qu'il n'avait pas de signe clinique d'hypothyroïdie. Cette hypothyroïdie apparait fortuitement dans un contexte de SSTV probablement primaire et a bien évolué après substitution hormonale. Dans le syndrome d'hypersécrétion hormonale, la manifestation la plus fréquente est l'hyperprolactinémie qui peut être due à un micro adénome et/ou à l'interruption du frein dopaminergique hypothalamique [9]. Du point de vue du diagnostic en imagerie médicale, la radiographie latérale du crâne peut révéler une selle de taille normale ou être agrandi. La selle vide typique montre une «montgolfière symétrique», c'est-à-dire une cavité régulière et incurvée de la selle. Actuellement, elle est devenue obsolète et insuffisante. Des tomographies informatisées montreront que la fosse pituitaire est occupée en grande partie par la substance du LCR ou la densité de l'eau plutôt que par une glande normale. L'imagerie par résonance magnétique (IRM), méthode de choix dans l'exploration de la région hypophysaire, peut facilement confirmer le diagnostic d’un sella vide. Sur les images IRM sagittales T1, l'extension du LCR dans la selle est facilement identifiable et la glande restante est comprimée le long du sol. La position centrale typique de l’infundibulum est un signe utile d'une selle vide qui permet d’éliminer une lésion kystique dans la région supra-sellaire. L’IRM démontrera que la sella est remplie de LCR et que l'infundibulum traverse l'espace, excluant ainsi une masse kystique. C'est ce que l’on appelle le signe de l'infundibulum [7]. L'accessibilité à l'IRM étant encore limitée à cause de sa non disponibilité mais aussi et surtout à cause de son cout élevé dans nos milieux, nous avons posé notre diagnostic par le scanner cérébral.

## Conclusion

Le SSTV est arachnoidocèle intrasellaire souvent de découverte fortuite caractérisé par une élévation transitoire ou constante de la pression intracrânienne. Il est souvent rencontré chez les obèses. Il doit être considéré comme diagnostic différentiel chez les patients ayant des maux de tête non spécifiques, en particulier les obèses.

## Conflits d’intérêts

Les auteurs ne déclarent aucun conflit d'intérêts.
